# Myoinositol vs. Metformin in Women with Polycystic Ovary Syndrome: A Randomized Controlled Clinical Trial

**DOI:** 10.3390/metabo12121183

**Published:** 2022-11-26

**Authors:** Pernille Ravn, Freja Gram, Marianne S. Andersen, Dorte Glintborg

**Affiliations:** 1 Department of Gynecology and Obstetrics, Odense University Hospital, 5000 Odense, Denmark; 2 Open Patient Data Explorative Network (OPEN), University of Southern Denmark, 5000 Odense, Denmark; 3 Department of Endocrinology, Odense University Hospital, 5000 Odense, Denmark

**Keywords:** PCOS, myoinositol, metformin, HOMA-IR, cycle length

## Abstract

Polycystic ovary syndrome (PCOS) is associated with insulin resistance. Few randomized controlled trials (RCT) compared myoinositol (MI) with metformin (MET) regarding insulin resistance in PCOS. This was an open-label six-month RCT in women with PCOS (*n* = 45) with interventions MI 4 g/day or MET 2 g/day. Primary outcome was the homeostasis model assessment of insulin resistance (HOMA-IR). Secondary outcomes were fasting glucose, weight, cycle length, lipids, testosterone, adverse effects, quality of life, and depression scores. Median age was 26 years. Body mass was index was 34.4 kg/m^2^. HOMA-IR was unchanged during MI (*p* = 0.31) and MET (*p* = 0.11) (MI vs. MET, *p* = 0.09). Median fasting glucose changed +0.2 mmol/L during MI (*p* < 0.001) and −0.1 mmol/L during MET (*p* = 0.04) (MI vs. MET *p* < 0.001). Median weight changed −2.3 kg during MI (*p* = 0.98) and −6.1 kg during MET (*p* < 0.001) (MI vs. MET, *p* = 0.02). Median cycle length decreased nine days during MI (*p* = 0.03) and 13 days during MET (*p* = 0.03) (MI vs. MET, *p* = 0.93). High-density lipoprotein (HDL) changed +0.1 mmol/L during MET (*p* = 0.04) (MI vs. MET, *p* = 0.07). All other blood parameters and scores of quality of life and depression remained unchanged during MI and MET (all *p* > 0.06) (MI vs. MET, all *p* > 0.27). Adverse effects appeared in four women during MI and 16 women during MET (MI vs. MET, *p* = 0.001). In conclusion, there was no effect on the metabolic outcomes during MI, but positive effects on fasting blood glucose, weight, and HDL during MET. The effect on cycle length was comparable during MI and MET. Adverse effects were less frequent during MI.

## 1. Introduction

Polycystic ovary syndrome (PCOS) is an endocrine disorder that affects more than 10% of women of reproductive age [1,2]. PCOS is characterized by irregular menstrual cycles, hirsutism, and polycystic ovaries [2]. Central obesity and insulin resistance are central elements in the etiology of PCOS [3,4]. The risk of type 2 diabetes is about four times increased in women with PCOS [5]. Furthermore, psychological health is often impaired [6,7].

Myoinositol (MI) is a naturally occurring insulin sensitizer found in citrus fruits and beans [8]. MI is a second messenger in the follicle-stimulating hormone (FSH) signaling pathway [9], and MI deficiency is related to ovulatory dysfunction in PCOS [9]. Ovulation frequency increased during MI treatment in women with PCOS where pregnancy was attempted [10,11]. Additionally, positive effects of MI on insulin resistance, triglycerides, and testosterone were reported in studies of women with PCOS [12,13], and these study results suggest that MI could be applied in PCOS outside fertility settings. The effect of MI on psychological health is undetermined.

Metformin (MET) is a well-known insulin sensitizer [14,15]. MET is prescribed in PCOS due to positive effects on insulin resistance and cycle length [16]. Body mass index (BMI) declines on average 1.25 kg/m^2^ during MET in obese women with PCOS [17]. Supplement of MET to lifestyle intervention has no additional effect on insulin sensitivity in PCOS, which infers that weight loss is the main mechanism for decreased insulin resistance during MET [18]. The mechanism for improved menstrual cycle during MET treatment is related to decreased insulin resistance [15] including a direct effect on ovarian insulin sensitivity [19]. Quality of life (QoL) was unchanged during MET in women with PCOS [20,21]. Adverse effects during MET frequently lead to treatment interruption [15,22], and in particular, gastrointestinal (GI) side effects are common.

The effect of MI vs. MET monotherapy on symptoms of PCOS varied in randomized controlled trials (RCT) conducted in women with PCOS not attempting pregnancy [23,24,25,26,27,28,29,30]. A recent meta-analysis of RCTs comparing MI and MET (among other comparisons of insulin sensitizers) concluded that MET was more effective than MI in terms of the homeostasis model assessment of insulin resistance (HOMA-IR), but the data have been few regarding the comparisons of other clinical parameters of PCOS [31]. GI side effects have been described to be less common during MI than during MET [32], but controlled studies on MI vs. MET in women with PCOS outside fertility settings are few. No study has been performed on women from North European countries and no previous study included measures of insulin resistance, cycle length, side effects, and QoL in a setup resembling the usual outpatient clinical management of PCOS.

The present aim was to examine MI vs. MET monotherapy in Danish women with PCOS not attempting pregnancy in an open-label, six-month RCT.

## 2. Materials and Methods

The participants were recruited through the PCOS outpatient clinic, Department of Gynecology and Obstetrics in collaboration with the Department of Endocrinology, Odense University Hospital (OUH), Denmark. Inclusion criteria were PCOS diagnosed according to the Rotterdam criteria [2] and age 18–50 years. Exclusion criteria were other causes of oligomenorrhea and/or hirsutism including abnormal values of prolactin, thyroid stimulating hormone, or 17-hydroxy-progesterone, postmenopausal values of FSH (>25 IE/L), and type 1 or 2 diabetes mellitus.

Pausing was required for MET and oral contraceptive pills for at least one and three months, respectively, before study entry. No woman was on MI before the study. Use of barrier contraception or copper intrauterine device during the study was optional. Women getting pregnant during the trial were excluded.

The study design was a 6 month open-label RCT. Randomization was conducted through the digital platform Research Electronic Data Capture (REDCap^®^) housed in The Unit for Good Clinical Practice, Odense Patient Explorative Network (OPEN) OUH. The sachets of MI contained 2 mg MI and 200 mg folic acid (Inofolic^®^, BiO4U Ltd., Dublin, Ireland) and were administered as one dose twice daily. Dose titration of MET was one tablet of 500 mg (Metformin, Actavis, TEVA, Tel Aviv, Israel) twice daily for two weeks followed by two tablets twice daily. Empty packages were counted at the final visit. Examinations were performed at the baseline and six months. Telephone interviews were scheduled after three months to record compliance and adverse effects.

### 2.1. Outcomes

The primary study outcome was HOMA-IR calculated as fasting serum insulin × fasting blood glucose/22.5. Secondary outcomes were fasting glucose, serum lipids, anthropometric measures (weight, BMI, waist, and hip circumference), Ferriman–Galwey (FG) score, cycle length, gonadothrophins, testosterone, anti-Müllerian hormone (AMH), and scores of QoL and depression as well as adverse effects.

### 2.2. Blood Samples and Assays

Blood samples were drawn in the morning after an overnight fast in the follicular phase in menstruating women, but arbitrarily in women with more than three months of secondary amenorrhea.

Serum insulin levels were analyzed by an electrochemiluminescence immunoassay (ECLIA) (Cobas e 801, Roche Diagnostics, Basel, Switzerland). Intra-assay coefficient of variation (CV) was 3.2–3.7% and inter-assay CV was 4.2–4.6%. Fasting plasma glucose was analyzed by ultraviolet hexokinase analysis-based absorption photometry (Cobas 8000, Roche Diagnostics, Basel, Switzerland). CV was 2.4%. Plasma high-density lipoprotein (HDL) cholesterol, total cholesterol, and triglycerides were analyzed by enzymatic colorimetric analysis-based absorption photometry (Cobas 8000, Roche Diagnostics, Basel, Switzerland), and low-density lipoprotein (LDL) cholesterol was calculated using the Friedewald equation. CVs were 1.3–2.3%. Sex hormone binding globulin (SHBG) was analyzed by chemiluminescence in a sandwich assay (Immulite 2000 XPI, Siemens Healthineers, Erlangen, Germany). CV was 4.7%. Plasma total testosterone was analyzed by liquid chromatography tandem mass spectrometry (Thermo Fischer Scientific, Waltham, MA, USA). CV was 3.3%. Free testosterone was calculated based on the Vermeulen equation with a standard albumin value of 4.3 g/dL [33]. AMH, FSH, LH, and estradiol were analyzed on an ECLIA (Cobas e 411 and 8000, Roche Diagnostics, Basel, Switzerland). CVs were 5.7–8%.

### 2.3. Menstrual Cycle, Ovarian Ultrasound

Menstrual cycle length was self-reported in a menstrual cycle calendar. Polycystic ovaries (PCO) were determined by vaginal ultrasound as the preference. Abdominal ultrasound was chosen by some women for personal reasons.

### 2.4. FG Score

Hirsutism was evaluated by the modified FG scoring system [34].

### 2.5. Adverse Effects and Pregnancies

Adverse effects and pregnancies were registered throughout the study using a personal diary.

### 2.6. Questionnaires

Two self-reportable, validated, Danish versions of the generic questionnaires were administered at the baseline and six months. The Short Form Health Survey (SF-36) explores eight areas: physical functioning, role limitations due to physical health, role limitations due to emotional health, energy/fatigue, emotional well-being, social functioning, pain, and general health. Each category is scored on a scale from 0–100, where higher scores indicate better functioning. The ICD10 Major Depression Inventory (MDI) is a rating scale to measure the degree of depression, where <20 is no depression and 50 indicates severe depression.

### 2.7. Statistics

For power calculation, the type 1 error was set to 0.05, and the type 2 error was set to 0.1. The six-month RCT of MI and MET in insulin-resistant women with PCOS was used as a reference for the power calculation [23]. Assumption of a non-inferiority margin of 1 standard deviation indicated 18 patients in each group. Drop-out rate of approximately 10% was expected, which concluded the inclusion of 20 patients in each group. The Mann–Whitney U test was used to compare the baseline data and Δ-values (6–0 months) MI vs. MET. The Wilcoxon Signed-Rank test was used to test within-group changes in the MI and MET groups. Pearson correlation analysis was used to test the correlation between Δ-values of HOMA-IR and weight. Calculations were performed with Stata/SE 17.0 software. A *p*-value of at least 0.05 was considered statistically significant. Data are presented as the median and interquartile range (IQR) (25%; 75%).

### 2.8. Ethical Approval

Participants gave their written informed consent after oral and written information. The Local Ethics Committee and Danish Medicines Agency approved the study, approval code S-20160188, date of approval 27 February 2017. The trial complied with the Declaration of Helsinki and standards for Good Clinical Practice. The Clinical Trials Register (https://www.clinicaltrialsregister.eu/ctr-search/search?query=2016-004506-34+ (accessed on 26 January 2017)), registration number (EudraCT) is 2016-004506-34. The date of registration was 11 November 2016. The first patient was enrolled 25 April 2017. The end of the trial was 29 October 2021.

## 3. Results

**Figure 1:**
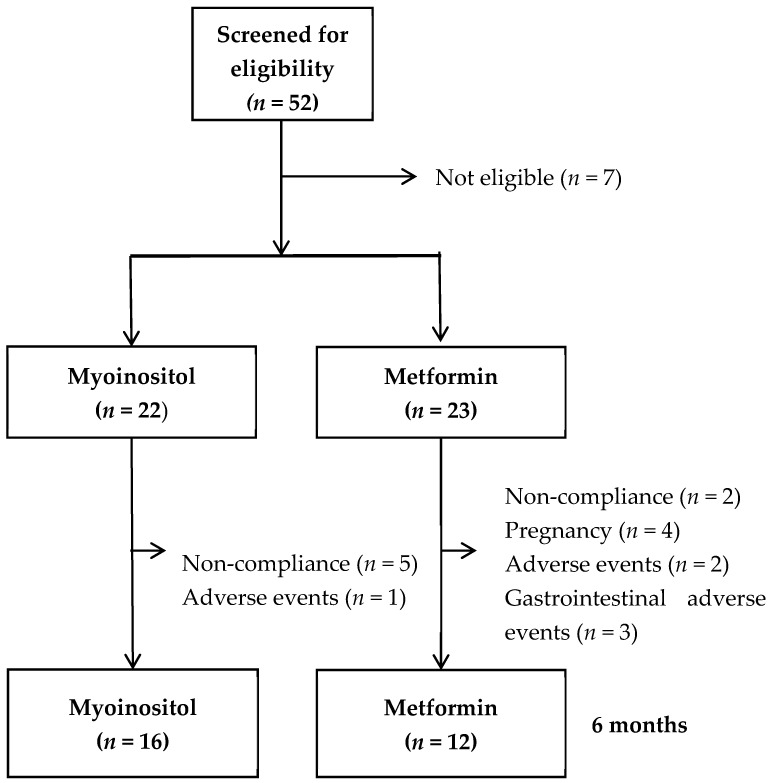
Flowchart of the study.

In all, 52 women with PCOS were screened for study inclusion (Figure 1), 45 women were included, and 28 women completed the study (MI *n* = 16, MET *n* = 12) (Figure 1). At the baseline, women in the two study groups were comparable regarding all study parameters (Table 1).

### 3.1. Metabolic Changes

HOMA-IR was unchanged within the MI and MET groups and Δ-values were comparable between MI vs. MET (*p* = 0.09) (Table 1). Median fasting glucose increased in the MI group, 0.2 mmol/L (*p* < 0.001), and decreased in the MET group, −0.1 mmol/L (*p* = 0.04), and the Δ-values were different between MI vs. MET (*p* < 0.001). Median HDL cholesterol was unchanged in the MI group, but increased in the MET group, 0.1 mmol/L (*p* = 0.04), and the Δ-values were comparable between MI vs. MET (*p* = 0.07). For all other lipids, the median within-group changes were non-significant (NS) and the Δ-values were comparable.

### 3.2. Anthropometric Values

The weight and BMI did not change significantly in the MI group, but decreased 6.1 kg (*p* < 0.001) and 2.4 kg/m^2^ (*p* < 0.001), respectively, in the MET group and Δ-values were different between MI vs. MET (both *p* = 0.02) (Table 1). Waist and hip circumferences were unchanged in both treatment groups and Δ-values were comparable between MI vs. MET (*p* ≥ 0.11). HOMA-IR Δ-values and weight Δ-values were correlated, but only significantly for the MI and MET groups combined, r = 0.51 (MI) (*p* = 0.08), r = 0.54 (MET) (*p* = 0.13), and r = 0.53 (MI and MET combined) (*p* = 0.01).

### 3.3. Cycle Length

The reduction in cycle length was 9 days in the MI group (*p* = 0.03) and 13 days in the MET group (*p* = 0.03), and the Δ-values were comparable between MI vs. MET (*p* = 0.92) (Table 1). 

### 3.4. FG Score

The FG scores did not change significantly during MI and MET, and the Δ-values were comparable between MI vs. MET (*p* = 0.49) (Table 1).

### 3.5. Gonadotrophins, Sex Hormones, and AMH

LH did not change significantly during MI and MET, but Δ-values were different between MI vs. MET (*p* = 0.04) due to a nonsignificant increase of LH during MI (*p* = 0.15) and a nonsignificant decrease of LH during MET (*p* = 0.16) (Table 1). For the other parameters, the median within-group changes were non-significant (NS) and the Δ-values between MI vs. MET were comparable.

### 3.6. Adverse Effects, Non-Completion, and Pregnancy

Adverse effects were registered in four women during MI: headache (*n* = 2), irregular menstruation (*n* = 1), and GI side effects (*n* = 1). One woman withdrew from the study due to adverse effects: irregular menstruation (a change from amenorrhea, which was subjectively perceived as unwanted).

Adverse effects were registered in 16 women during MET: headache (*n* = 1), mood swing (*n* = 1), increased body hair (*n* = 2), and GI side effects (*n* = 12). Five women withdrew from the study due to adverse effects: GI side effects (*n* = 3), headache (*n* = 1), and mood swings (*n* = 1); MI vs. MET regarding adverse effects (*p* < 0.01), MI vs. MET regarding GI side effects (*p* < 0.01), and MI vs. MET regarding withdrawal (*p* < 0.001).

Reasons for noncompletion were low compliance (MI, *n* = 5 and MET, *n* = 2), pregnancy (MI *n* = 0 and MET *n* = 4), and adverse effects (MI, *n* = 1 and MET *n* = 5), MI vs. MET (*p* < 0.01) (Figure 1). Non-completers had lower baseline general health scores than the study completers, a median value of 45 vs. 62 (*p* = 0.01), lower emotional well-being score, 64 vs. 72 (*p* = 0.03), and higher MDI score, 27 vs. 12 (*p* = 0.02).

### 3.7. Quality of Life

Scores of QoL and depression did not change significantly in any of the groups and the Δ-values were comparable (Table 2).

## 4. Discussion

In the present RCT, we compared MI and MET in a well-characterized Danish group of women with PCOS outside a fertility setting. The main finding was that MI did not improve the metabolic study outcomes since HOMA-IR, Hb1Ac, insulin, and weight remained unchanged and fasting glucose even increased during MI treatment. In contrast, MET had beneficial effects on fasting glucose, HDL cholesterol, and weight. MI and MET had a comparable and positive effect on the menstrual cycle length. The present study design included medical intervention without lifestyle intervention, which allowed us to observe the isolated effect of MI vs. MET on the study outcomes and adverse events.

Unchanged HOMA-IR [24] and weight [24,25,26,28] during MI were in accordance with some previous RCTs on MI vs. MET. In contrast, HOMA-IR decreased during MI to a comparable extent as during MET in other studies [23,25,27,29,30] without concomitant life style intervention like in our study. The most pronounced reduction during MI in HOMA-IR (0.4–0.6) and BMI (2.0 kg/m^2^) was found in the Italian six month RCT [23]. Remaining studies reported smaller reductions of HOMA-IR (0.1–0.2) and BMI (0.2–0.5 kg/m^2^) [29,30] or a decrease in HOMA-IR without concurrent weight loss [25] during MI vs. MET, indicating a subtle effect of MI. None of the studies investigated the effect of MI vs. MET in a placebo controlled setup, but several uncontrolled studies showed the beneficial effects of MI on both HOMA-IR and weight in South European and Middle Eastern study populations, as described in a recent narrative review [9]. Although current evidence is mostly negative, it thus remains possible that MI has a minor, but subtle effect on insulin sensitivity and weight, and the long-term effect of MI on the risk of type 2 diabetes in PCOS needs further investigation.

MET was associated with a 6.8% decrease in BMI in our study, which matched the results from a recent meta-analysis [15]. Furthermore, HOMA-IR tended to decrease during MET, but the change did not reach statistical significance, which was possibly related to type 2 error. This is substantiated by our findings that changes in HOMA-IR correlated with Δ-weight for the combined study groups. This and previous RCTs of MI vs. MET monotherapy [23,24,25,26,27,28,29,30] confirmed the beneficial effects of MET on metabolic symptoms of PCOS and support that MET is superior to MI regarding metabolic risk factors in PCOS.

Cycle length decreased to a comparable extent during MI and MET; nine and 13 days, respectively. Decreased cycle length during MI and MET corresponded to results from previous RCTs of MI vs. MET monotherapy in PCOS [23,24]. Combined MI and MET treatment improved the cycle length to a greater extent (3 days) than MET monotherapy [35], which could suggest different mechanisms of MI and MET on ovarian function. Importantly, our results supported that the effect of MI on menstrual cycle was independent of body weight, sex hormones, AMH, and insulin, which were unchanged during MI. In accordance, MI has been shown to be a second messenger in the FSH signaling pathway, which implies a direct effect of MI on ovulatory function [9]. Consistently, MI improved clinical pregnancy rates during in vitro fertilization [9]. A weight loss of 5–10% induced positive effects on the menstrual cycle in PCOS [36,37]. Improved menstrual cycle length in the MET group could thus be a result of a direct effect of MET on the ovarian insulin sensitivity and an indirect effect of improved insulin sensitivity through weight loss [38].

Adverse effects were generally milder and less frequent during MI than during MET. Most remarkably, GI side effects were reported by one woman only during MI, whereas GI side effects were reported by 14 women during MET. Three women withdrew from the study due to GI side effects during MET. Our results correspond to the findings of the RCTs that reported results on adverse effects during MI vs. MET [23,24]. In terms of consistency, MI is reported to have few or no adverse effects, whereas MET treatment in PCOS frequently leads to GI side effects, which compromise adherence to treatment [32]. A meta-analysis showed that the relative risk of adverse effects during MET vs. MI was 5.2 [32], and one review reported that drop-out rates due to GI side effects during MET in PCOS can be up to 50% [14]. This led us to conclude that MI can be applied in women with PCOS and oligomenorrhea where MET is not tolerated. Interestingly, non-completion was highest during MI, which could be related to the limited clinical effect.

Scores of QoL and depression were unchanged during MI and MET in the present study. In contrast, one previous 12-week RCT showed significantly improved QoL during MI and MET in PCOS, but the effect was small and could be of minor clinical relevance [28]. Baseline scores of QoL in all areas except physical functioning and general health were lower than the average scores in healthy women of similar age [39], which supported low QoL in obese women with PCOS. Low scores of SF-36 and QoL are related to weight and hirsutism in PCOS [39,40] and low QoL is associated with weight in obese women without PCOS [41]. Weight and hirsutism was unchanged during MI in the present study, whereas weight decreased and FG score was unchanged during MET. We previously reported unchanged SF-36 and PCOS visual analog scale (PCOS-VAS) during 12 months of treatment with oral contraceptive pills and MET, irrespective of decreased hirsutism and weight loss during treatment [21]. These observations support that other mechanisms could be related to low QoL in PCOS. Depressive symptoms could be mediated by hyperactivity of the HPA axis [42] and hyperinflammation [43] in PCOS, and studies are needed regarding the optimal treatment of impaired QoL in PCOS.

Strengths and limitations apply to the present study. The strength of the present study was the RCT design with study outcomes covering the metabolic and hormonal phenotypes of PCOS and quality of life. The study was powered to determine non-inferiority of MI vs. MET regarding HOMA-IR, but results regarding secondary metabolic outcomes were consistently in favor of MET. The study population had no specific requirements for participation other than PCOS (i.e., the population was representative of women with PCOS attending a gynecologic outpatient clinic). The study design resembled average outpatient management including telephone contact after three months. Drop-out rate was high, which could be related to the lower scores of QoL and depression in non-completers. Psychosocial support could have increased study completion, but would not resemble the resources of the usual health care systems.

## 5. Conclusions

In conclusion, HOMA-IR and weight were unchanged during MI whereas MET had beneficial effects on weight, fasting blood glucose, and HDL cholesterol. Cycle length decreased to a similar extent during MI and MET. Adverse effects were less frequent during MI vs. MET. MI may be considered as a second line treatment of oligomenorrhea in PCOS, if MET is not tolerated and oral contraceptives are not requested.

## Figures and Tables

**Table 1:** Baseline characteristics and changes after 6 months in the myoinositol and metformin group.

Intervention	Myoinositol (*n* = 16)			Metformin (*n* = 12)			
Time	Baseline	6 Months	Δ *p*	Baseline	6 Months	Δ *p*	Δ vs. Δ *p*
HOMA-IR (pmol mmol L^−2^)	28.5 (19.6;45.6)	33.7 (27.1;49.6)	0.31	30.9 (24.9;32.4)	18.8 (14.3;29.6)	0.11	0.09
Insulin (pmol/L)	126 (85;190)	119 (78;219)	0.38	117 (112;143)	80 (65;121)	0.21	0.13
Glucose (mmol/L)	5.2 (5.0;5.4)	5.4 (5.3;5.7)	0.00	5.3 (5.1;5.7)	5.2 (4.8;5.3)	0.04	0.00
HbA1c (mmol/mol)	32.0 (30.0;36.0)	33.0 (31.0;34.0)	0.18	33.5 (32.0;35)	32.0 (31.5;33.5)	0.47	0.12
Triglycerides (mmol/L)	1.2 (1.0;1.4)	1.3 (1.0;1.6)	0.09	1.0 (0.9;1.2)	1.0 (0.8;1.5)	0.81	0.25
HDL (mmol/L)	1.2 (1.0;1.3)	1.2 (1.1;1.3)	0.92	1.3 (1.2;1.4)	1.4 (1.2;1.6)	0.04	0.07
LDL (mmol/L)	2.9 (2.4;3.2)	2.9 (2.5;3.1)	0.44	3.1 (2.6;3.3)	2.9 (2.6;3.3)	0.93	0.64
Cholesterol (mmol/L)	4.6 (4.1;4.9)	4.7 (4.1;5.1)	0.46	4.8 (4.3;5.2)	4.8 (4.2;5.3)	0.69	0.42
Age (years)	25 (22;34)	-		27 (24;33)	-		-
Weight (kg)	96.7 (84.5;107.6)	94.4 (84.9;107.0)	0.98	99.8 (86.4;108.9)	93.7(81.7;106.8)	0.00	0.02
BMI (kg/m^2^)	34.2 (30.9;37.2)	34.5 (29.9;36.8)	0.96	35.2 (31.0;39.8)	32.8 (29.8;38.5)	0.00	0.02
Waist (cm)	103 (93;108)	100 (93;104)	0.31	99 (92;106)	100 (90;105)	0.41	0.62
Hip (cm)	122 (108;125)	119 (110;125)	0.84	123 (114;129)	119 (110;128)	0.06	0.11
Cycle length (days)	45 (35;175)	36 (32;60)	0.03	47 (35;82)	34 (28;37)	0.03	0.92
FG score	6 (0;17)	8 (3;12)	0.79	6 (2;12)	6 (2;12)	0.40	0.49
SHBG (nmol/L)	30 (18;46)	30 (14;37)	0.78	35 (27;57)	38 (29;44)	0.88	0.92
Free testosterone (nmol/L)	0.032 (0.025;0.038)	0.028 (0.025;0.045)	0.89	0.027 (0.019;0.035)	0.021 (0.017;0.028)	0.06	0.70
Total testosterone (nmol/L)	1.5 (1.1;1.7)	1.2 (1.0;1.8)	0.39	1.5 (1.3;1.8)	1.2 (0.9;1.7)	0.10	0.28
AMH (pmol/L)	37.7 (25.1;51.8)	36.0 (31.6;44.4)	0.96	38.9 (33.9;78.4)	38.3 (25.8;57.0)	0.46	0.74
FSH (IU/L)	5.8 (4.6;7.0)	5.4 (4.9;7.0)	0.67	5.4 (3.3;6.0)	5.7 (4.3;6.6)	0.26	0.26
LH (IU/L)	8.9 (6.2;11.8)	12.0 (9.4;16.0)	0.15	12.5 (8.2;16.0)	10.4 (5.0;13.5)	0.16	0.04
Estradiol (nmol/L)	0.19 (0.14;0.41)	0.18 (0.15;0.25)	0.16	0.26 (0.17;0.70)	0.19 (0.13;0.29)	0.08	0.34

Values are shown as medians (IQR, interquartile range, 25%; 75%) BMI: body mass index, HOMA-IR: homeostatic model assessment for insulin resistance, HbA1c: hemoglobin A1c, HDL: high-density lipoprotein, LDL: low-density lipoprotein, FG: Ferriman–Gallwey, AMH: Anti-Müllerian hormone, FSH: follicle stimulating hormone, LH: luteinizing hormone, SHBG: sex hormone binding globulin. Δ *p*: *p*-value for Δ value (6–0 months), Δ vs. Δ *p*: *p*-value for comparison of the Δ values.

**Table 2:** Baseline characteristics and changes after 6 months in the myoinositol and metformin group.

Intervention	Myoinositol (*n* = 14)			Metformin (*n* = 12)			
Time	Baseline	6 Months	Δ *p*	Baseline	6 Months	Δ *p*	Δ vs. Δ *p*
SF-36							
Physical functioning	90 (75;95)	90 (80;95)	0.14	90 (85;95)	95 (85;100)	0.16	0.78
RPH	88 (50;100)	100 (50;100)	0.57	100 (75;100)	100 (50;100)	0.29	0.25
REP	100 (0;100)	100 (33;100)	0.58	100 (50;100)	100 (17;100)	0.28	0.29
Energy/fatigue	50 (35;60)	53 (30:65)	0.59	40 (25;65)	48 (30;73)	0.61	0.22
Emotional well-being	74 (60;84)	80 (52;84)	0.57	72 (58;84)	70 (52:84)	0.32	0.31
Social functioning	59 (45;80)	69 (38;80)	0.92	53 (36;73)	64 (40;79)	0.43	0.55
Pain	58 (45;90)	68 (55;90)	0.61	74 (68;95)	69 (68;90)	0.52	0.50
General health	65 (50;80)	63 (35;75)	0.25	58 (38;80)	68 (38;78)	0.42	0.17
MDI							
Score	11 (9;20)	10 (7;22)	0.92	13 (5;29)	15 (6;27)	0.87	1.0

Values are shown as medians (IQR, interquartile range, 25%; 75%). SF-36: Short Form Health Survey, RPH: role limitations due to physical health, REP: role limitations due to emotional problems, MDI: Major Depression Inventory. Δ *p*: *p*-value for Δ value (6–0 months), Δ vs. Δ *p*: *p*-value for comparison of the Δ values.

## Data Availability

Data are stored at the Open Patient Data Explorative Network (OPEN). Data are available in a publicly accessible repository: The data presented in this study are openly available in Open Patient Data Explorative Network (OPEN), Odense, Denmark. Bonafide researchers can apply to use the dataset by applying to open@rsyd.dk.
